# Immune infiltration and a necroptosis-related gene signature for predicting the prognosis of patients with cervical cancer

**DOI:** 10.3389/fgene.2022.1061107

**Published:** 2023-01-06

**Authors:** Xuewei Xing, Yanan Tian, Xuan Jin

**Affiliations:** ^1^ The First Clinical Medical College, School of Medicine, Nanchang University, Nanchang, China; ^2^ Department of Assisted Reproduction, The First Affiliated Hospital of Nanchang University, Nanchang, China; ^3^ Postgraduate Union Training Base of Jinzhou Medical University, Xiangyang No 1 People’s Hospital, Hubei University of Medicine, Xiangyang, China; ^4^ Key Laboratory of Zebrafish Modeling and Drug Screening for Human Diseases of Xiangyang City, Department of Obstetrics and Gynaecology, Xiangyang No. 1 People’s Hospital, Hubei University of Medicine, Xiangyang, China

**Keywords:** cervical cancer, necroptosis, prognosis, immune microenvironment, TCGA

## Abstract

**Background:** Cervical cancer (CC), the fourth most common cancer among women worldwide, has high morbidity and mortality. Necroptosis is a newly discovered form of cell death that plays an important role in cancer development, progression, and metastasis. However, the expression of necroptosis-related genes (NRGs) in CC and their relationship with CC prognosis remain unclear. Therefore, we screened the signature NRGs in CC and constructed a risk prognostic model.

**Methods:** We downloaded gene data and clinical information of patients with cervical squamous cell carcinoma and endocervical adenocarcinoma (CESC) from The Cancer Genome Atlas (TCGA) database. We performed functional enrichment analysis on the differentially expressed NRGs (DENRGs). We constructed prognostic models and evaluated them by Cox and LASSO regressions for DENRGs, and validated them using the International Cancer Genome Consortium (ICGC) dataset. We used the obtained risk score to classify patients into high- and low-risk groups. We employed the ESTIMATE and single sample gene set enrichment analysis (ssGSEA) algorithms to explore the relationship between the risk score and the clinical phenotype and the tumor immune microenvironment.

**Results:** With LASSO regression, we established a prognostic model of CC including 16 signature DENRGs (*TMP3*, *CHMP4C*, *EEF1A1*, *FASN*, *TNF*, *S100A10*, *IL1A*, *H1.2*, *SLC25A5*, *GLTP*, *IFNG*, *H2AC13*, *TUBB4B*, *AKNA*, *TYK2*, and *H1.5*). The risk score was associated with poor prognosis in CC. Survival was lower in the high-risk group than the low-risk group. The nomogram based on the risk score, T stage, and N stage showed good prognostic predictive power. We found significant differences in immune scores, immune infiltration analysis, and immune checkpoints between the high- and low-risk groups (*p* < 0.05).

**Conclusion:** We screened for DENRGs based on the TCGA database by using bioinformatics methods, and constructed prognostic models based on the signature DENRGs, which we confirmed as possibly having important biological functions in CC. Our study provides a new perspective on CC prognosis and immunity, and offers a series of new targets for future treatment.

## 1 Introduction

Cervical cancer (CC) is one of the most common cancers in women, and it is the fourth leading cause of cancer death (Vu et al., 2018). Globally, there are an estimated 530,000 new cases and 270,000 deaths each year. Today, preventive human papillomavirus (HPV) vaccination for CC is available worldwide, but more than a quarter of patients with CC still die each year due to a severe lack of medical supplies in many developing countries (Small et al., 2017). Despite the multidisciplinary approach of surgery combined with chemotherapy that has been applied to patients with CC, their prognosis remains unsatisfactory, making the search for an effective therapeutic target an urgent issue (Ghasemi et al., 2019).

Cell death is an important component in maintaining homeostasis in an organism, and resistance to cell death is usually the cause of tumor formation. Cell death can be divided into two types: necrosis and apoptosis. In recent years, a novel type of cell death has been identified that differs from necrosis and apoptosis, namely necroptosis, which is mechanistically and morphologically similar to apoptosis and necrosis (Gong et al., 2019). Necroptosis, a complementary mode of apoptotic failure, is a type of programmed cell death that is activated by caspase-independent signaling pathways, mainly by the receptor-interacting protein kinase 1 and 3 (RIPK1/RIPK3)/mixed lineage kinase domain-like protein (MLKL) complex (Quarato et al., 2016). Necroptosis is thought to play a key role in cancer progression and metastasis, and some studies have identified necroptosis-related genes (NRGs) as possible biomarkers of cancer prognosis (Gong et al., 2019). MLKL has recently been identified as a downstream component of RIPK3, a key factor in tumor necrosis factor (TNF)-induced necroptosis, and as a prognostic biomarker in CC (Ruan et al., 2015). Necroptosis also plays an important role in tumor immunology and cancer immunotherapy, where it is involved in triggering CD8^+^ T cell–driven antitumor immunity (Sprooten et al., 2020). RIPK3, a regulator of necroptosis in tumor cells, also serves as a novel predictive marker for cancer immunotherapy personalization (Smola, 2016). Fibroblasts in the tumor microenvironment (TME) induce a robust immune response through necroptosis and initiate transduction through nuclear factor κB (NF-κB) signaling (Yatim et al., 2015). Considering its important role in cancer biology and antitumor immunity, necroptosis has emerged as a new target for bypassing cell death resistance and modulating antitumor immunity and tumor therapy in oncological treatment. Induction of necroptosis by pharmacological intervention is emerging as a promising tool for multiple anti-apoptotic cancer cells. RETRA has been shown to play a role in CC treatment as a drug-induced necroptosis anticancer agent by selectively inducing necroptosis in CC cells through phosphorylation of the structural domains of RIPK1/RIPK3 and MLKL (Mohanty et al., 2022). However, to date, few studies have investigated the significance of NRGs in the prognosis and immunotherapy of CC.

In this study, we screened NRGs as prognostic biomarkers for CC using The Cancer Genome Atlas (TCGA) database and constructed an associated risk prediction model based on 16 signature DENRGs. We comprehensively analyzed the role of NRGs in CC and highlighted their prognostic and immunotherapeutic potential for CC. Analysis of immune infiltration, TME, immune checkpoints, mutations, and clinicopathological features revealed significant differences between the high- and low-risk CC groups. Our study provides accurate prognostic predictions and effective immunotherapy strategies for patients with CC.

## 2 Materials and methods

### 2.1 Data collection

We extracted transcriptome profiles, clinical characteristics, and tumor mutation data (simple nucleotide variation) of patients with cervical squamous cell carcinoma and endocervical adenocarcinoma (CESC) from the TCGA database (https://portal.gdc.cancer.gov/). We collected NRGs from the following Gene Ontology (GO) terms: necroptotic process (GO:0070266), execution phase of necroptosis (GO:0097528), necroptotic signaling pathway (GO:0097527), negative regulation of necroptotic process (GO:0060546), positive regulation of necroptotic process (GO:0060545), regulation of necroptotic process (GO:0060544), ripoptosome assembly involved in necroptotic process (GO:1901026), negative regulation of programmed necrotic cell death (GO:0062099), positive regulation of programmed necrotic cell death (GO:0062100), programmed necrotic cell death in response to starvation (GO:0097385), regulation of mitochondrial membrane permeability involved in programmed necrotic cell death (GO:1902445), and regulation of programmed necrotic cell death (GO:0062098). We identified 651 genes associated with necrotizing apoptosis *via* GeneCards. A published study proposed 159 genes (Zhang et al., 2022). After removing the duplicated genes involved in the above GO terms, we had a total of 749 NRGs for the downstream analysis.

### 2.2 Identification and functional analysis of differentially expressed NRGs in CC

We identified differentially expressed genes (DEGs) between 306 CC and three adjacent control samples by using the “DESeq2” R package with |log_2_FC| >1 and adjusted *p*-value <0.05 as criteria (Waardenberg and Field, 2019). We obtained differentially expressed NRGs (DENRGs) by overlapping DEGs with the 749 NRGs. We used the “ClusterProfiler” R package to screen significantly enriched GO terms and Kyoto Encyclopedia of Genes and Genomes (KEGG) pathways of DENRGs with the threshold of an adjusted *p*-value <0.05. Furthermore, we uploaded DENRGs to the STRING database (https://string-db.org/) to investigate their interactions.

### 2.3 Construction and verification of the risk score model in CC

According to the expressions of prognostic DENRGs and coefficients, we calculated the risk score of each patient in the training set with the following formula: ∑(coefficient × gene expression). According to the median value of the risk score, we divided patients in the training set into high- and low-risk groups. We analyzed the overall survival of high- and low-risk groups with Kaplan-Meier analysis. To evaluate the accuracy of risk score model, we plotted receiver operating characteristic (ROC) curves using “survivalROC” in R. We used the International Cancer Genome Consortium (ICGC) dataset as the validation group to verify the above results. Moreover, to test the reliability of the risk score model, we conducted similar analyses in the validation set.

### 2.4 Construction of the nomogram to predict prognosis of CC

To determine independent prognostic factors for CC patients, we used clinical characteristics (age, sex, and TNM stage) and the risk score for univariate and multivariate Cox regression analysis. Then, we incorporated independent prognostic factors to construct the nomogram to predict the 1-, 3-, and 5-year survival of patients with CC. We plotted calibration curves to evaluate the performance of the nomogram.

### 2.5 Exploration of the mechanisms underlying necroptosis-related CC

To explore the potential mechanisms of prognostic DENRGs in regulating CC, we performed the following analyses. 1) We compared the risk score among different subgroups stratified by T stage (T1, T2, T3, T4), N stage (N0, N1), M stage (M0, M1), and grade (G1, G2, G3, G4) using the Wilcoxon or Kruskal–Wallis test to investigate the relationship between risk score and the progression of CC. 2) We downloaded GO and KEGG reference gene sets from the MSigDB database (https://www.gsea-msigdb.org/gsea/msigdb/) to perform gene set enrichment analysis (GSEA). We identified significantly enriched GO terms and KEGG pathways between the low- and high-risk score with an adjusted *p*-value <0.05. 3) We calculated the immune and stromal score of each patient with the ESTIMATE algorithm, and then determined the correlations between the risk score and immune/stromal scores. 4) We downloaded a 28 immune cell gene sets from The Cancer Imaging Archive (TCIA) database. We calculated the single sample GSEA (ssGSEA) scores for the different types of immune cells in each sample by using the “GSVA” package in R to compare the differences in immune infiltration levels between samples from the high- and low-risk groups (Cheng et al., 2021). We used the Wilcoxon test to analyze difference.

### 2.6 Analysis of the TME score, tumor mutation burden, and immune checkpoint molecules

1) We predicted the proportion of infiltrating stromal and immune cells in tumor tissue using the “estimate” package in R, based on ssGSEA. We generated the stromal score, the immune score, and the ESTIMATE score (Ke et al., 2021). 2) We downloaded mutation data from the TCGA database, selecting the TCGA-ESCA project, simple nucleotide variation as the data category, VarScan2 Variant Aggregation as the workflow type, and used masking. We used “maftools” in R to calculate the mutation load for each sample. We evaluated the mutations in the 16 genes used to establish the prognosis. 3) We extracted the expression of 47 immune checkpoint molecules from the training set expression matrix and compared their expression differences between the high- and low-risk groups using the Wilcoxon test. We screens 27 differentially expressed immune checkpoint–related genes between the high- and low-risk groups using an adjusted *p*-value <0.05 as the screening criterion.

### 2.7 Statistical analysis

We assessed the difference in overall survival between the high- and low-risk groups by the Kaplan-Meier method and log-rank test. We determined the predictive accuracy of the risk model by determining the area under the ROC curve (AUC). We used R version 4.0.0 for all analysis. We considered significant differences as *p* < 0.05 unless otherwise specified.

## 3 Results

### 3.1 One hundred eighty DENRGs are associated with CC

**FIGURE 1:**
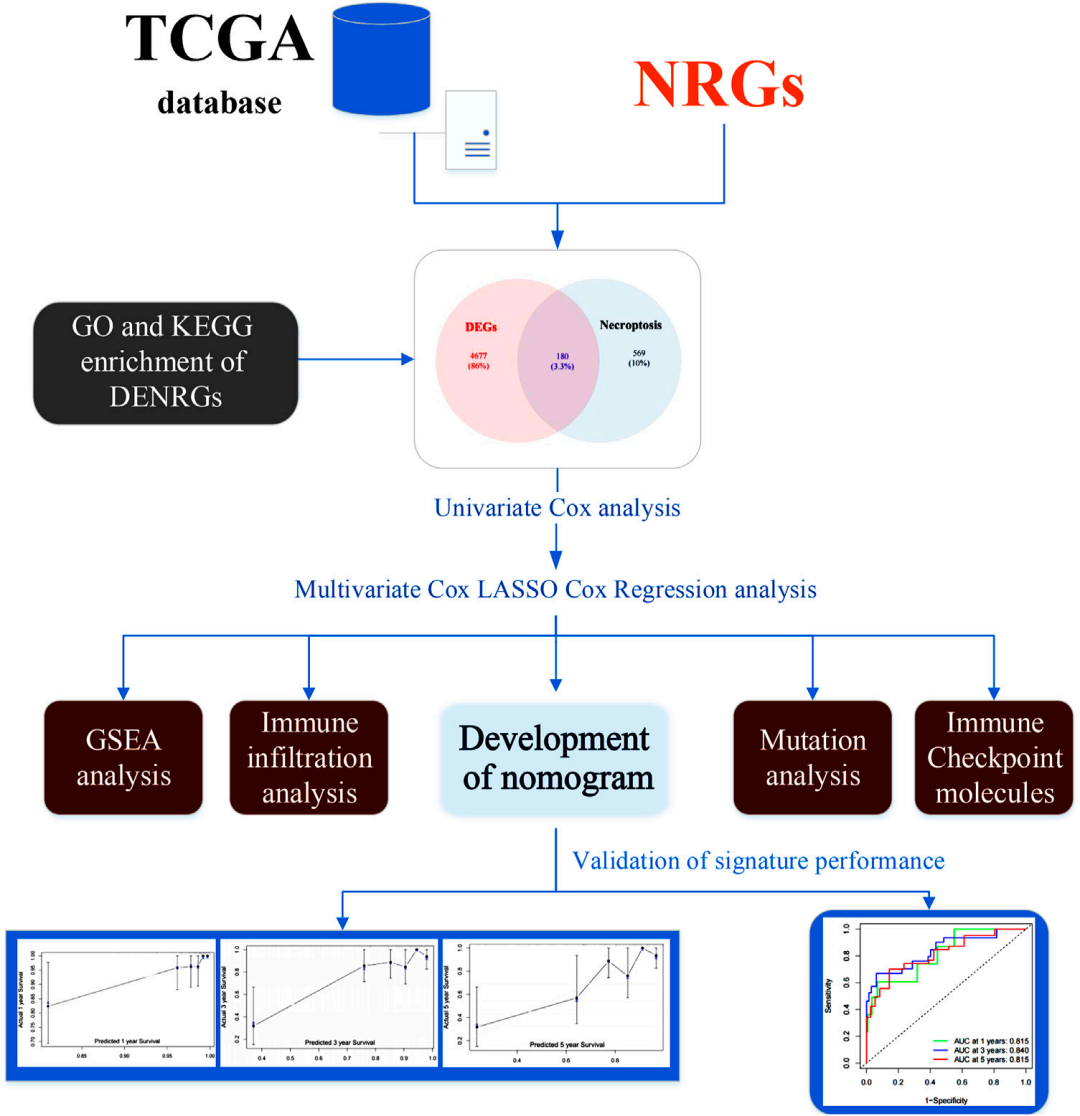
The flow chart of this study. Abbreviations: DENRGs, differentially expressed necroptosis-related genes; GO, Gene Ontology; GSEA, gene set enrichment analysis; KEGG, Kyoto Encyclopedia of Genes and Genomes; NRGs, necroptosis-related genes.

**FIGURE 2:**
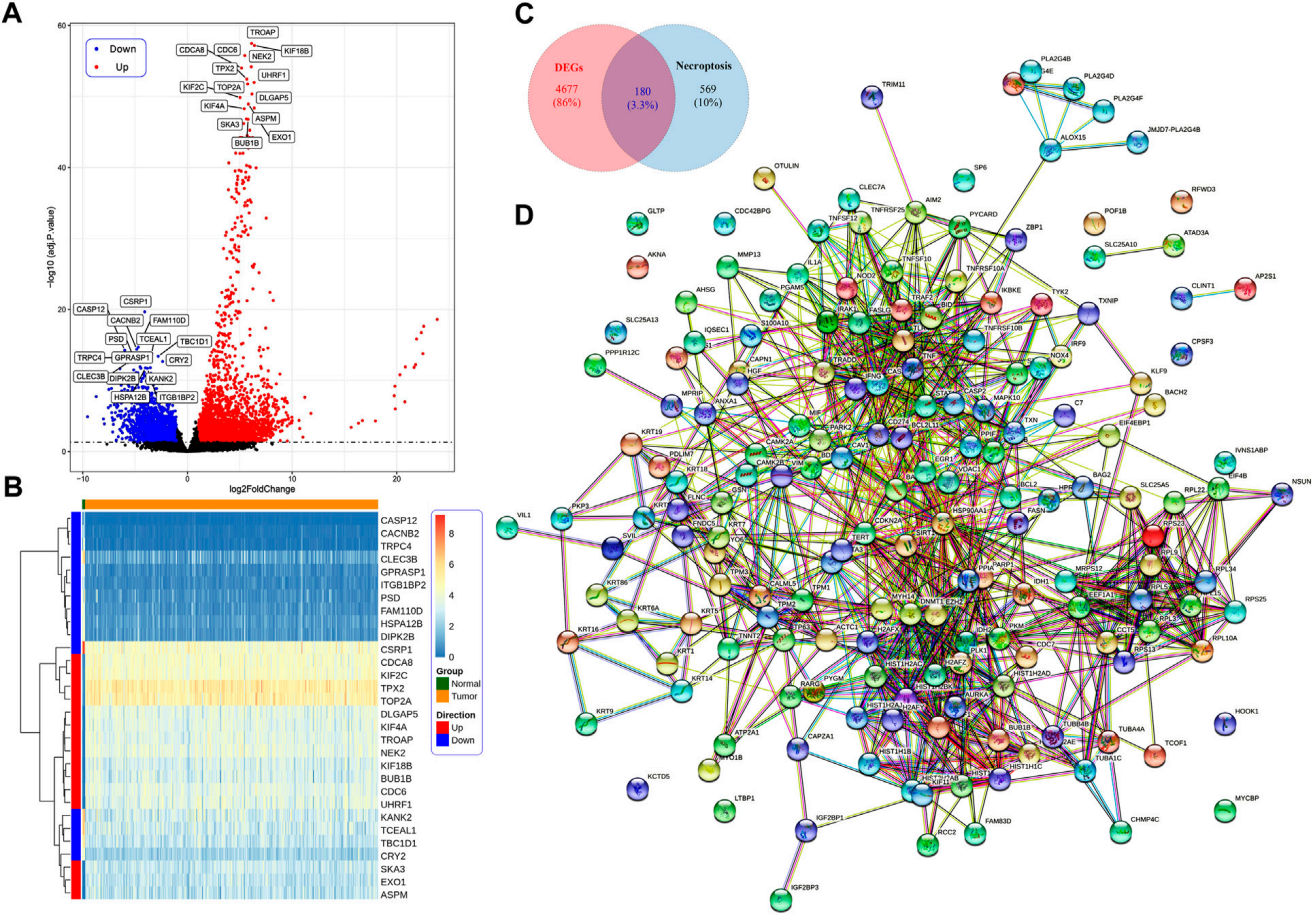
**(A)** Identification of differentially expressed necroptosis-related genes (DENRGs). Identification of differentially expressed genes (DEGs) in cervical cancer (CC) tumor tissue and normal control tissue based on a volcano plot. **(B)** The expression of the top 15 upregulated and downregulated genes in CC presented in a heatmap. **(C)** The expression of 180 DENRGs between the tumor and normal groups. **(D)** Protein–protein interaction network of the 180 DENRGs.

The study flow chart is presented in Figure 1. We identified 4,857 DEGs between the CC and control samples, including 2,998 upregulated and 1,859 downregulated genes (Figure 2A). We identified 749 NRGs from GeneCards and the literature; the list is shown in Table S1. The top 15 upregulated and top 15 downregulated genes are shown in a heatmap (Figure 2B); the logFC of the top 20 NRGs is shown in another heatmap (Supplementary Figure S1). A volcano plot of NRGs is presented in Figure S2. After overlapping DEGs with NRGs, we identified 180 genes as DENRGs (Figure 2C). The list of 180 DENRGs is shown in Table S2. In addition, we constructed a protein–protein interaction (PPI) network, in which we observed the interplay among most DENRGs (Figure 2D).

### 3.2 Functional enrichment of DENRGs

**FIGURE 3:**
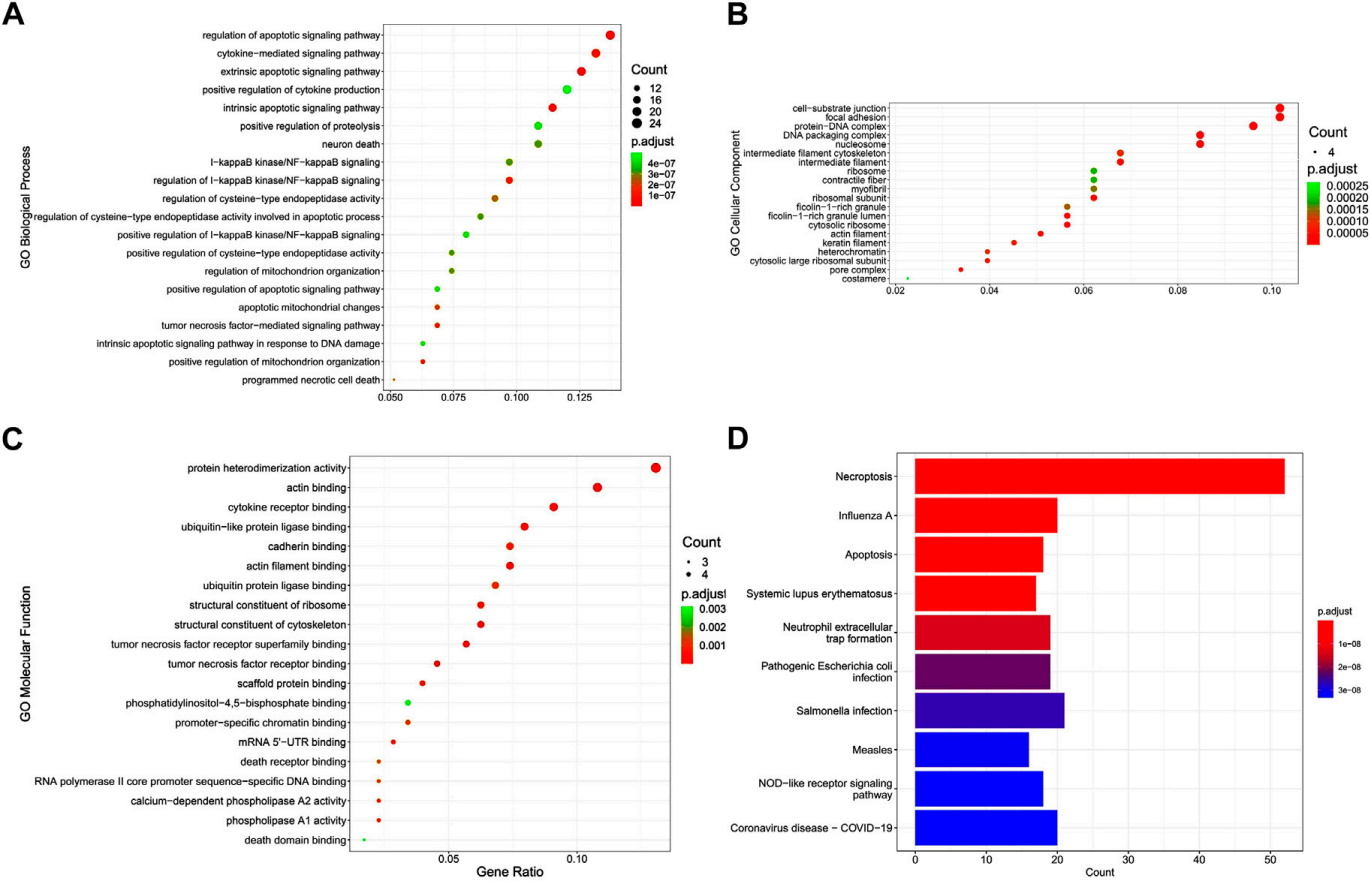
Gene Ontology (GO) and Kyoto Encyclopedia of Genes and Genomes (KEGG) enrichment analysis of differentially expressed necroptosis-related genes (DENRGs). GO enrichment of DENRGs for **(A)** biological processes, **(B)** cellular components, and **(C)** molecular functions. **(D)** KEGG enrichment of DENRGs.

Functional analysis showed that DENRGs are mainly enriched in GO terms and KEGG pathways relevant to necroptosis and apoptosis. In biological processes, DENRGs are mainly enriched in cell death and related receptor signaling pathways. In cellular components, DENRGs are mainly enriched in DNA damage repair related pathways. In molecular functions, DENRGs are enriched in pattern recognition receptor activity pathways (Figures 3A–C). Moreover, KEGG analysis revealed that these DENRGs are mainly enriched in necroptosis, influenza A, and apoptosis (Figure 3D).

### 3.3 Construction and validation of DENRG-related prognostic signature

**FIGURE 4:**
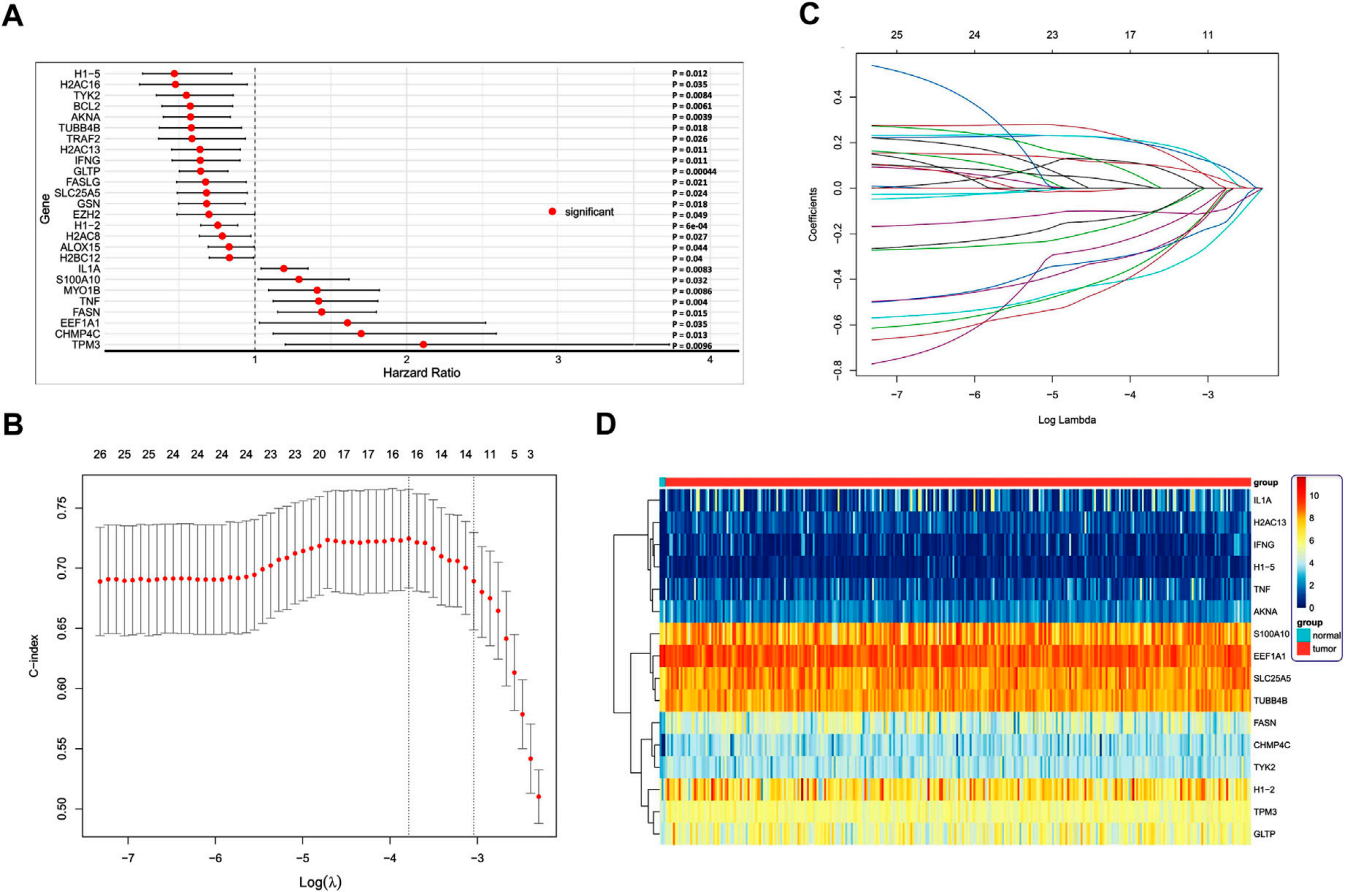
**(A)** Cox regression analysis to identify differentially expressed necroptosis-related genes (DENRGs) related to biochemical recurrence-free survival. Univariate Cox regression identified 26 DENRGs significantly associated with prognosis (*p* < 0.05). **(B)** Screening for genes that can be used independently for prognostic risk prediction using the best LASSO model parameter *λ*. **(C)** Variable number change. **(D)** Heatmap of the expression of the 16 DENRGs in the high- and low-risk groups.

We evaluated the prognostic value of 16 DENRGs in CC by univariate Cox and LASSO regressions. By univariate Cox regression, we found 26 DENRGs (*TPM3*, *CHMP4C*, *EEF1A1*, *FASN*, *TNF*, *MY O 1B*, *S100A10*, *IL1A*, *H2BC12*, *ALOX15*, *H2AC8*, *H1.2*, *EZH2*, *GSN*, *SLC25A5*, *FASLG*, *GLTP*, *IFNG*, *H2AC13*, *TRAF2*, *TUBB4B*, *AKNA*, *BCL2*, *TYK2*, *H2AC16*, and *H1.5*) to be significantly related with the survival of patients with CC (Figure 4A). To obtain a more robust prognostic signature, we input those 26 DENRGs into the LASSO algorithm. We narrowed the prognostic signature down to 16 DENRGs (*TPM3*, *CHMP4C*, *EEF1A1*, *FASN*, *TNF*, *S100A10*, *IL1A*, *H1.2*, *SLC25A5*, *GLTP*, *IFNG*, *H2AC13*, *TUBB4B*, *AKNA*, *TYK2*, and *H1.5*) (Figures 4B,C). Then, we calculated the DENRG-related prognostic as: [the expression of *TPM3* × 0.102505 + expression of *CHMP4C* × 0.189606 + expression of *EEF1A1* × 0.033772 + expression of *FASN* × 0.192809 + expression of *TNF* × 0.203929 + expression of *S100A10* × 0.007325 + expression of *IL1A* × 0.103649 + expression of *H1.2* × (−0.10396) + expression of *SLC25A5* × (−0.11957) + expression of *GLTP* × (−0.38244) + expression of *IFNG* × (−0.19587) + expression of *H2AC13* × (−0.10861) + expression of *TUBB4B* × (−0.31664) + expression of *AKNA* × (−0.27736) + expression of *TYK2* × (−0.27591) + expression of *H1.5* × (−0.34445)]. These 16 prognostic DENRGs were expressed abnormally in the CC samples compared with the normal samples (Figure 4D).

**FIGURE 5:**
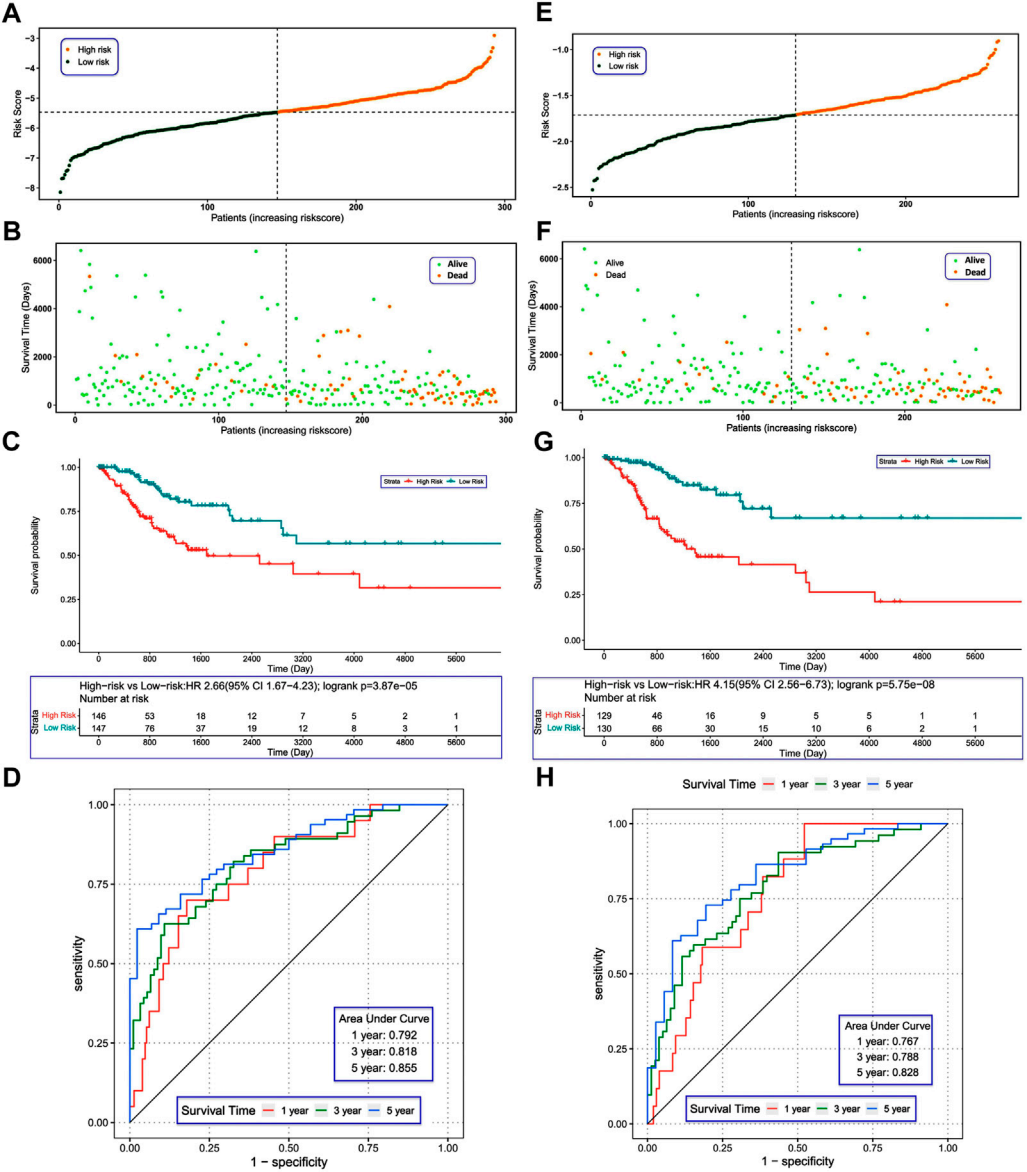
**(A)** Construction and evaluation of a risk model. Distribution of risk scores in the training set. **(B)** Survival status of patients in the training set. **(C)** Kaplan–Meier plot of the training set (*p* < 0.05). **(D)** Receiver operating characteristic curves showing the 1-, 3-, and 5-year predictive efficiency of the risk score. **(E)** Distribution of risk scores in the validation set. **(F)** Survival status of patients in the validation set. **(G)** Kaplan–Meier plot of the validation set (*p* < 0.05). **(H)** The area under the receiver operating characteristic curves in the validation set for 1-, 3-, and 5-year survival.

According to the median risk score, we divided the patients with CC in the TCGA training set into high- and low-risk groups (Figure 5A). As the risk score increased, we observed more dead patients (Figure 5B). The high-risk group had worse survival compared with the low-risk group (Figure 5C). The AUC of the ROC curves were 0.792, 0.818, and 0.855 for 1-, 3-, and 5-year survival, respectively (Figure 5D), suggesting that the risk score model had good performance in predicting the prognosis of patients with CC. Furthermore, we tested the risk score model in the ICGC validation set and obtained similar results (Figures 5E–G). The AUC of ROC curves in the validation set were 0.767, 0.788 and 0.828 for 1-, 3-, and 5-year survival (Figure 5H), further demonstrating the reliability of the DENRG-related prognostic signature in predicting the survival of patients with CC.

### 3.4 The development of a DENRG-related nomogram

**FIGURE 6:**
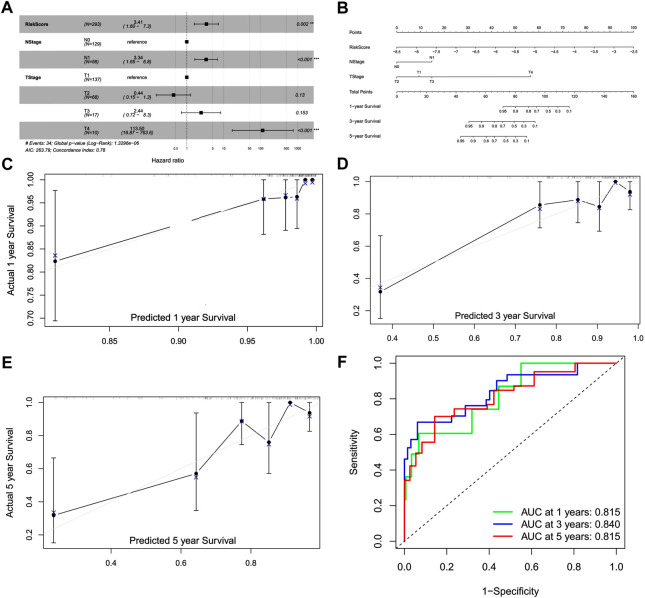
Construction of a prognostic model and nomogram. **(A)** Forest plot of the multivariate Cox model. **(B)** Nomogram for the prognostic model. **(C)** Calibration curve for 1-year survival. **(D)** Calibration curve for 3-year survival. **(E)** Calibration curve for 5-year survival. **(F)** Receiver operating characteristic curve for 1-, 3-, and 5-year survival.

We performed univariate and multivariate analyses with independent prognostic factors in CC, including the risk score, T stage, N stage (Figures S3, 6A). We established the nomogram based on prognostic factors to predict the 1-, 3-, and 5-year survival of patients with CC (Figure 6B). Calibration curves showed that the predicted overall survival was close to the actual overall survival (Figures 6C–E). The AUC values for 1-, 3-, and 5-year survival of the nomogram model were 0.815, 0.840, and 0.815, respectively, indicating the good performance of the nomogram (Figure 6F).

### 3.5 Risk score and tumor stage

**FIGURE 7:**
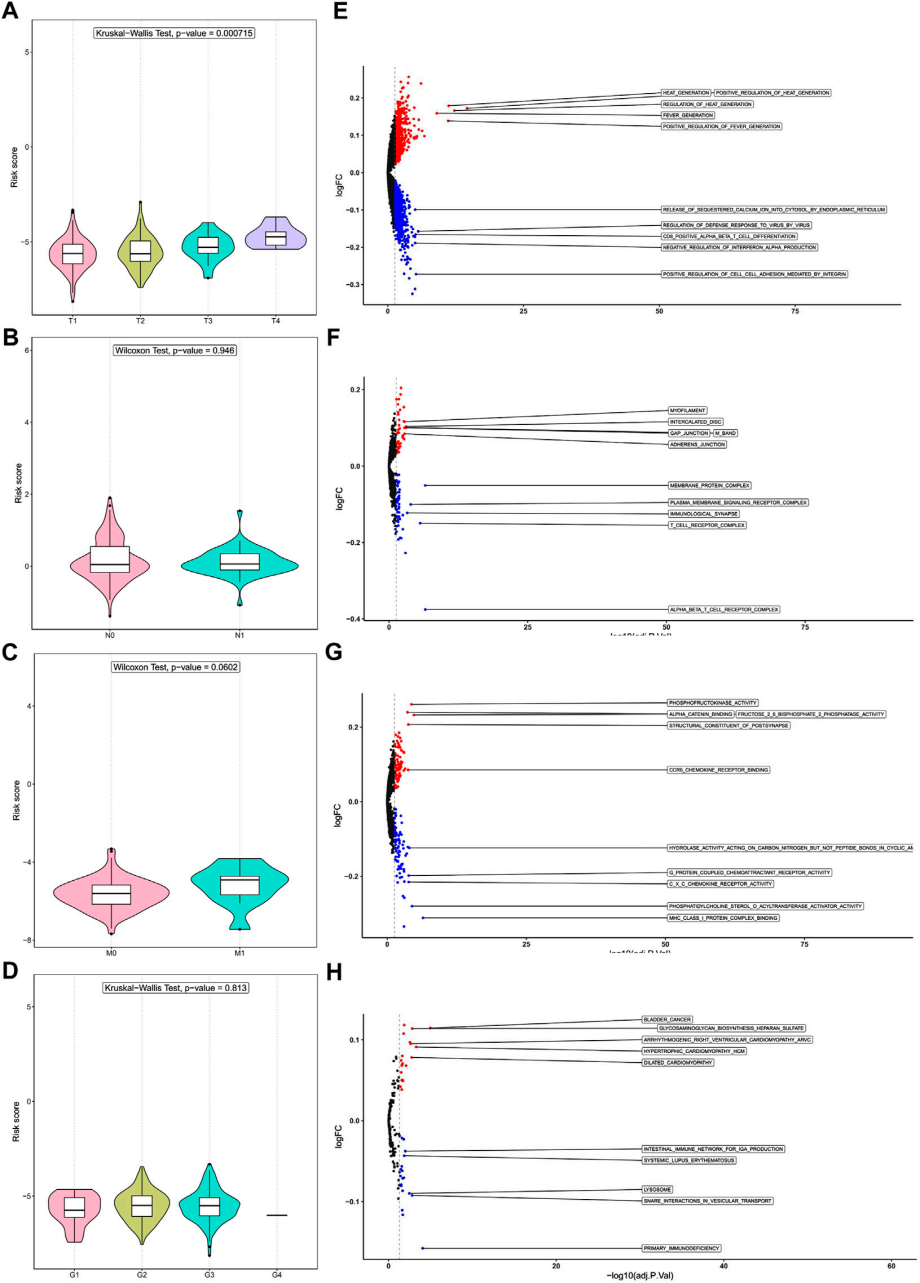
Risk score and tumor stages and gene set enrichment analysis (GSEA). Difference in risk score among **(A)** T stages, **(B)** N stages, **(C)** M stages, and **(D)** grades. Differences in pathway enrichment in tumors of patients in the high- and low-risk groups: **(E)** Gene Ontology (GO) analysis of biological processes, **(F)** GO analysis of cell components, and **(G)** GO analysis of molecular functions. **(H)** Kyoto Encyclopedia of Genes and Genomes (KEGG) enrichment analysis.

To further evaluate the utility of prognostic DENRGs, we compared the risk scores among different groups divided by T stage, N stage, M stage, and grade. Interestingly, we noticed that the risk score increased with the progression of CC, and patients with T4 CC had the highest risk scores (*p* < 0.05). However, the risk score was not significantly changed in other stages and grades (*p* > 0.05) (Figures 7A–D). These results indicate that prognostic DENRGs play important roles in the T stage metastasis and malignancy degree of CC, which may further affect the survival of patients with CC.

### 3.6 Gene set variation analysis between high- and low-risk groups

We performed GSVA to unveil the potential molecular mechanisms and to identify biological processes. For GO biological process, cellular component, and molecular function analysis, the pathways enriched in the high-risk group include cell junctions, phosphorylation, and other related pathways; the pathways enriched in the low-risk group include those related to viral defense, T-cell differentiation, cell adhesion, cytokine receptors, and protein coupling (Figures 7E–G). Among the KEGG pathways, the high-risk group was significantly enriched in pathways related to glycosaminoglycan biosynthesis, while the low-risk group was significantly enriched in pathways related to immunity and lysosomes (Figure 7H). These results suggest that prognostic DENRGs may regulate the development and progression of CC *via* cell proliferation.

### 3.7 Differences in immune-related outcomes between the high- and low-risk groups

**FIGURE 8:**
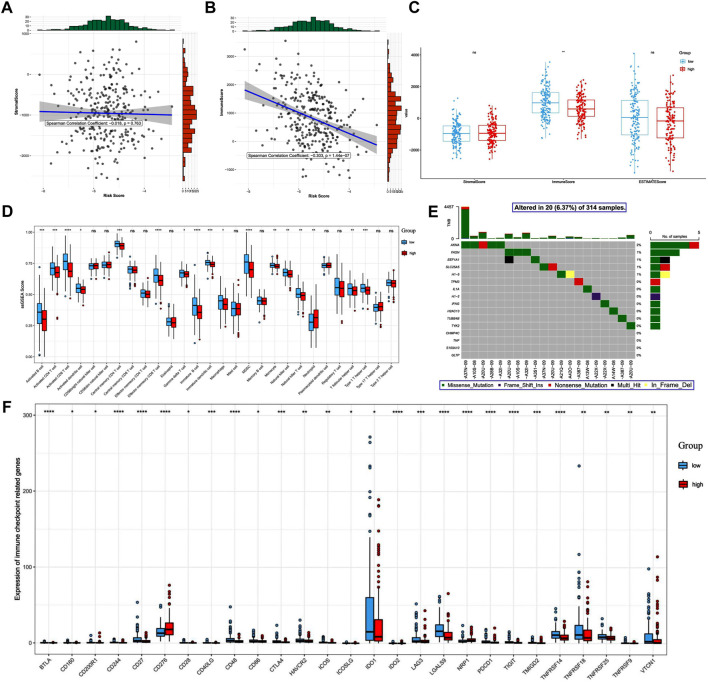
Differences in immune-related outcomes between the high-risk and low-risk groups. Risk and ESTIMATE scores: **(A)** correlation between the risk score and the stromal score. **(B)** Correlation between the risk score and the immune score. **(C)** Differences in tumor microenvironment scores between the high- and low-risk groups. **(D)** Boxplot of differences in the degree of immune cell infiltration. **(E)** Mutation map of prognosis-related genes in patients with cervical cancer. **(F)** Differential expression of immune checkpoint molecules.

Increasing evidence has demonstrated the important role of the immune microenvironment in the outcome of CC. Thus, we explored whether NRGs could modulate the immune microenvironment of patients with CC. We found that the risk score was not correlated with stromal score (*p* > 0.05, Figure 8A). In addition, although the risk score was significantly correlated with immune score, it was weak (*p* < 0.01, r = -0.303, Figure 8B). The TME score analysis showed that the immune score was significantly higher in the low-risk group than in the high-risk group (*p* < 0.01, Figure 8C). Furthermore, we compared the immune infiltration between patients in the low- and high-risk groups and observed that the infiltration levels of activated B-cell, activated CD4^+^ T-cell, activated CD8^+^ T-cell, activated dendritic cells, central memory CD4^+^ T-cell, effector memory CD4^+^ T-cell, immature B-cell, immature dendritic cells, natural killer T-cell, and natural killer T-cell, were different (Figure 8D). Figure 8E shows the mutation status of the 16 genes used to build the prognostic model.

## 4 Discussion

The occurrence and development of CC is a complex process regulated by multiple factors, and the incidence of CC has been trending younger in recent years (Torre et al., 2015; Small et al., 2017). Necroptosis is a newly discovered mode of programmed cell death and often used as an alternative when apoptosis induction is compromised. Necroptosis is involved in tumor proliferation and metastasis and is closely associated with the TME and tumor immunity (Su et al., 2015). In recent years, it has been shown that necroptosis enhances anti-tumor immunity in cancer therapy and could become an effective cancer treatment (Wu et al., 2022). Therefore, we developed an NRG characterization model to predict the prognosis of patients with CC, and we used the obtained risk score to group them into high- and low-risk groups for the analysis of clinical indicators, the ESTIMATE score, the tumor mutational load, immune checkpoint molecules, and immune infiltrating cells.

Both apoptosis and necroptosis are forms of programmed cell death, which is a natural barrier limiting the survival and propagation of malignant cells. The molecular mechanisms regulating necroptosis are closely related to the signaling cascades that control apoptosis and necrosis (Schmidt et al., 2015). MLKL is a prognostic biomarker for cervical squamous cell carcinoma and has recently been identified as a key RIPK3 downstream component of TNF-induced necroptosis (Ruan et al., 2015). Through the use of CC models, PolyIC-driven immunogenicity has been shown to be dependent on the necroptosis regulator RIPK3 in tumor cells, suggesting that RIPK3 could serve as a novel predictive marker for the personalization of cancer immunotherapy (Smola, 2016). Considering the above criteria, NRGs may be involved in necroptosis, necrosis, apoptosis, tumor immunity, and their interactions. Consistently, based on our GO, KEGG, and GSEA analyses, these DENRGs are involved in necroptosis, necrosis, apoptosis, and immune- and tumor-related pathways.

We identified 16 DENRGs, and they have been reported that are associated with necroptosis. *TPM3*, *CHMP4C*, *EEF1A1*, *FASN*, *TNF*, *S100A10*, and *IL1A* represent a high-risk score and poor prognosis, suggesting that these genes may be associated with the tumor process in patients with CC and appear to be pro-oncogenes. In contrast, *H1.2*, *SLC25A5*, *GLTP*, *IFNG*, *H2AC13*, *TUBB4B*, *AKNA*, *TYK2*, and *H1.5* are abundantly expressed in the low-risk group, suggesting that these genes may be oncogenes for CC. CHMP4C is highly expressed in CC tissues and cell lines and plays a role as a pro-oncogene in them (Lin et al., 2020). The EEF1A protein blocks apoptosis and facilitates viral replication and interacts with the E7 protein of human papillomavirus (HPV) 38 to participate in events related to incidence of CC formation-related events (Ghittoni et al., 2010). FASN plays a key role in tumor lipid metabolism and is associated with the tumor-associated phosphoinositide 3-kinase (PI3K)/AKT signaling pathway. Its overexpression is often associated with tumor progression and poor prognosis (Cao et al., 2017). FASN may be a potential therapeutic target for CC, and a FASN inhibitor (orlistat) reduces apoptosis triggered by CC cells (C-33A, ME-180, HeLa, and SiHa) in a time-dependent manner (Nascimento et al., 2022). S100A10 may have anti-apoptotic effects in cancer cells, interacting with Bad and impeding its pro-apoptotic activity (Bharadwaj et al., 2021). The S100A10 subunit promotes L2-mediated human papillomavirus infection, which is associated with the development of CESC (Taylor et al., 2018). Elevated expression of IL1A, a pleiotropic pro-inflammatory cytokine, is associated with poorer prognosis in CC through multiple complex mechanisms involving cell proliferation, apoptosis, angiogenesis, and the inflammatory microenvironment (Song et al., 2016). There is growing evidence that H1-2 has important functions in multiple cellular processes including apoptosis, autophagy, the cell cycle, and gene transcription. H1-2 acts as a signaling molecule to initiate apoptosis, and its deletion may lead to resistance to apoptosis in mice and tumor cells (Lai et al., 2021). Both SLC25A5 and GLTP are associated with good prognosis in CC (Qu et al., 2021). Colon cancer cells overexpressing GLTP (HT-29) exhibit RIPK-3-mediated MLKL phosphorylation, increased intracellular Ca^2+^, levels and induce cell death through necroptosis (Mishra et al., 2019). The CC-associated oncoprotein HPV E6 can downregulate AKNA and lead to cancer progression (Wang et al., 2020). In contrast, AKNA contributes to dysregulation of the cancer immune system and can serve as a genetic factor and biomarker of susceptibility to CC (Ramírez-González et al., 2021). This is consistent with our results that TYK2 is a protective gene in the prognostic model of necroptosis in CC (Ding et al., 2020). Both TNF and IFGN are triggers of necroptosis and can synergistically induce RIPK-dependent necroptosis (Hojo et al., 2019). MLKL was initially identified as a key mediator of TNF-induced necroptosis and can be used to assess the prognosis of patients with cervical squamous cell carcinoma, and TNF is also important for immune and cellular homeostasis in mammals (Ruan et al., 2015). Its role as a major regulator to balance cell survival, apoptosis, and necroptosis has been studied extensively in various cell types and tissues (Blaser et al., 2016). IFNG is an immune response gene and some of its single nucleotide polymorphisms (SNPs) are associated with cervical carcinogenesis and plays a decisive prognostic role in squamous cervical cancer (Chen et al., 2020). However, there are few reports on the role of *TPM3*, *H2AC13*, *TUBB4B*, and *H1.5* in CC, which will be an important direction for our future research.

Resistance to apoptosis is one of the characteristics of tumors, and therefore induction of cell death mechanisms other than apoptosis is emerging as a new cancer treatment strategy. Necroptosis mediates cancer-related immune responses by promoting interactions between cancer and immune cells through the release of damage-associated molecular patterns (DAMPs), cytokines, or chemokines in the TME (Sprooten et al., 2020). The TME plays an important role in the course of tumorigenesis, progression, and prognosis of CC. In this study, we found that risk scores were negatively correlated with immune scores, and different risk score groups showed different TME infiltration characteristics. In low-risk group, the abundance of CD8^+^ T-cell, CD4^+^ T-cell, and NK cells were increased. These immune cells have been widely reported as effector cells in the TME, and have a positive immune response to cancer cells (Litwin et al., 2021). Furthermore, tumor cells undergo necroptosis, which activates CD8^+^ T-cell to eliminate cancer cells and thus induce an anti-tumor immune response. Necroptosis-associated genes (EEF1A1, IFNG) can activate CD8^+^ T-cell (Guan et al., 2022). DAMP from necrotic tumor cells can induce strong anti-tumor CD8^+^ T-cell expression (Yatim et al., 2015). According to our results, it indicates that the lower the risk score, the better the immunity and prognosis of patients. In high-risk group, neutrophil was increased. Tumors can increase tumor cell proliferation by inducing the conversion of neutrophils into tumor-associated neutrophils and releasing inflammatory mediators (Demkow, 2021). Therefore, the patients with high risk score might have more severe inflammatory reaction, tumor proliferation and worse prognosis. Overall, we could roughly predict immunity of patients according to their risk score.

Immune checkpoints present an effective immunosuppressive mechanism in cancer, providing more effective treatment options to improve cancer survival. Immune checkpoint inhibitor (ICI) therapy is considered an effective treatment for CC (Mauricio et al., 2021). In our results, most immune checkpoint molecules—including *BTLA*, *CD27*, *CD28*, *CD86*, *CTLA4*, *ICOS*, *ID O 1*, *TIGIT*, *TNFRSF14*, *TNFRSF18*, *TNFRSF25*, *TNFRSF9*, and *VTCN1*—are highly expressed in the low-risk group. CTLA-4, CD28, BTLA, TIGIT, and ICOS belong to the immunoglobulin-associated receptor family and are responsible for various aspects of T-cell immune regulation. CTLA-4 is an immune checkpoint protein receptor that downregulates the immune system. CTLA-4 has been identified as a prognostic marker in CC, and CTLA-4 inhibitors CTLA-4 inhibitors in combination with radiation/chemotherapy may improve outcomes for patients with CC (Odiase et al., 2021). Blocking CTLA-4 allows the body to overcome HPV-driven immunosuppression associated with CC (Callahan and Wolchok, 2013). In addition to conventional ICIs targeting CTLA4, PD-1, and PD-L1, novel ICIs including agonists targeting BTLA, TIGIT, and the co-stimulatory receptor ICOS are increasingly being used in clinical therapy. IDO1 induces immunosuppression of T-cell by depleting l-tryptophan and kynurenine in the local TME, suppressing effector T-cell and over-activating regulatory T-cell (Jung et al., 2019). Blockade of IDO1 contributes to shrinkage of CC (Blocking IDO1 Helps Shrink Bladder, Cervical Tumors, 2018). In addition, members of the tumor necrosis factor receptor superfamily (TNFRSF) are present on T-cell and play a key role in T-cell development, survival, immune activation, and the anti-tumor immune response (Schaer et al., 2014). Combined with our results, patients in the high-risk group may be less sensitive to ICIs, and thus ICI treatment may be more effective in the low-risk group. Taken together, these immune checkpoint molecules may be explored as meaningful targets for CC, and the combination strategy of ICIs with radiation/chemotherapy offers a new direction for the future treatment of CC and may help to overcome resistance to radiation/chemotherapy and immunotherapy alone.

In conclusion, we have identified 16 NRGs that are significantly associated with CC prognosis. Our findings provide possible explanations for the different prognostic assessments of patients with CC and offer prospects for future studies on necroptosis as a therapeutic target for CC and exploration of new immunotherapeutic approaches.

## Data Availability

The datasets presented in this study can be found in online repositories. The names of the repository/repositories and accession number(s) can be found in the article/Supplementary Material.

## Abbreviations

AUC, area under the curve; CESC, Cervical squamous cell carcinoma and endocervical adenocarcinoma; DAMPs, Damage-associated molecular patterns; DENRGs, Differentially expressed necroptosis-related genes; GO, Gene Ontology; GSEA, Gene set enrichment analysis; GSVA, Gene set variation analysis; HPV, Human papillomavirus; ICIs, Immune checkpoint inhibitors; KEGG, Kyoto Encyclopedia of Genes and Genomes; NRGs, Necroptosis-related genes; PPI, Protein–protein interaction; ROC, receiver operating characteristic; SNPs, Single nucleotide polymorphisms; TCGA, The Cancer Genome Atlas; TNFRSF, Tumor necrosis factor receptor superfamily.
