# Supplementary material for: Immune infiltration and a necroptosis-related gene signature for predicting the prognosis of patients with cervical cancer
Source: Front Genet. 2023 Jan 6;13:1061107. doi: 10.3389/fgene.2022.1061107 (PMC9852722; doi:10.3389/fgene.2022.1061107)
Supplement: Supplementary file 1 [file Table1.DOCX]

| **749 NRGs** |
| --- |

| CFLAR | CAMK2G | H2AZ2 | CD40LG | RANBP2 | PLEC | SNRPF |
| --- | --- | --- | --- | --- | --- | --- |
| BIRC3 | SLC25A5 | H2AC7 | MIR155 | RPL11 | TUBA1B | SRSF6 |
| FAS | SLC25A6 | H2AZ1 | KIAA1191 | PRPS1 | XRN2 | SRSF4 |
| YBX3 | SLC25A31 | H2AC15 | TP63 | SLC25A1 | KRT2 | SRSF3 |
| CASP8 | PPID | H2AC6 | PADI4 | MYO6 | KRT9 | CLINT1 |
| CYLD | VDAC1 | H2AC13 | MIR22 | FLNC | NCL | CEP170 |
| BAX | VDAC2 | H2AC14 | MIR214 | HNRNPA2B1 | ELAVL1 | AMBRA1 |
| DNM1L | VDAC3 | H2AC16 | MIR101-2 | HNRNPA1 | IGF2BP3 | CPSF6 |
| TRPM7 | GLUD2 | H2AB2 | CTSD | IKBKE | AP2S1 | COPS2 |
| PYGL | GLUD1 | PPIA | CLEC7A | MYH14 | PAWR | CARD6 |
| ITPK1 | GLUL | PGLYRP1 | HPRT1 | IL4 | RPL26 | CAPN7 |
| ASAH1 | PYGM | CASP6 | FAP | NUP214 | RPL9 | ATXN2L |
| CAV1 | PYGB | UCHL1 | TRIM24 | PFKL | RPL4 | ADRM1 |
| PPIF | MAPK8 | TNIP1 | CHL1 | RIPK2 | RPL7 | LRRFIP2 |
| BIRC2 | MAPK10 | STING1 | TGFBR1 | UBE2D3 | PSMC4 | PREB |
| ARHGEF2 | MAPK9 | SERTAD1 | ACVR1B | TPM2 | RPS13 | PKP3 |
| FASLG | FTH1 | MAPK14 | CCL2 | UGDH | RPS23 | ZNF24 |
| ZBP1 | FTL | KLHDC10 | MEFV | TUFM | RPS26 | ZFP36 |
| ALKBH7 | PLA2G4E | GSK3B | DPEP1 | TUBB4A | RPS29 | YBX1 |
| RBCK1 | PLA2G4A | TNIP3 | AIM2 | TRAF6 | RPL10A | ZNF217 |
| RIPK3 | JMJD7-PLA2G4B | GJB1 | UBR2 | TET2 | RPS12 | ZC3HAV1 |
| RIPK1 | PLA2G4B | PTGES3 | GPX4 | COPB2 | RPL12 | GCC2 |
| TP53 | PLA2G4C | BRD4 | LAMP2 | ATF4 | SVIL | FAF2 |
| OGT | PLA2G4D | RB1 | MPRIP | EIF4EBP1 | SNRPE | HELQ |
| IPMK | PLA2G4F | DAPK1 | MTOR | CLTC | CAPZA1 | GTF3C3 |
| SLC25A4 | ALOX15 | FKBP1A | AFP | CALM2 | EIF4B | CDC42BPG |
| SPATA2 | CAPN1 | MIR425 | ACHE | LTBP1 | CUL4A | RPL17 |
| TLR3 | CAPN2 | EZH2 | NFAT5 | DDX5 | CNBP | RPS25 |
| FADD | SMPD1 | NFE2L2 | VIL1 | PPP1CB | BANF1 | RPS27L |
| MLKL | PYCARD | CD274 | CXCL1 | PRSS1 | AP2B1 | TNRC6B |
| NLRP6 | CASP1 | CXCL5 | MAPK1 | TUBA4A | MAP1LC3A | TWF1 |
| NUPR1 | IL1B | MIR29B1 | CBL | KRT19 | RNGTT | TANC2 |
| BOK | CHMP2A | MERTK | GSN | KRT6A | RPL3 | ALYREF |
| FZD9 | CHMP2B | TYRO3 | TBK1 | LGALS3 | PCBP1 | CPSF2 |
| PELI1 | CHMP3 | SIRT6 | RARG | FUS | PRPF8 | CPSF3 |
| MIR107 | RNF103-CHMP3 | NAT2 | CTSH | EMD | SLC25A10 | DCD |
| MIR103A1 | CHMP4B | SFTPA1 | CTSL | HSPA1A | TUBB6 | MGA |
| MIR101-1 | CHMP4A | FLOT1 | CTSS | GBE1 | IGF2BP1 | RAI14 |
| MIR221 | CHMP4C | FLOT2 | TNFRSF25 | MYO5A | HNRNPF | RPL38 |
| MIR485 | CHMP6 | PDCD6IP | DSTYK | PARK7 | HSPA1B | OTUD4 |
| TNF | VPS4B | FASN | IKBKB | RPS10 | IQSEC1 | PLEKHA5 |
| PGAM5 | VPS4A | CDK9 | CHUK | RPL35 | IL24 | TXNIP |
| MPG | CHMP1B | TIMM50 | BUB1B | S100A10 | MPP1 | WRNIP1 |
| TNFRSF1B | CHMP1A | SLC25A37 | IRAK1 | S100A4 | MYL6 | ZNF146 |
| TRAF2 | CHMP5 | PPP1R3G | MAPK3 | CALM1 | IVNS1ABP | LIMCH1 |
| HAT1 | CHMP7 | MIR7-1 | TAB2 | CCT5 | MVP | LACTB |
| SIRT2 | IL1A | NFKBIA | EIF2AK3 | AFG3L2 | PDIA4 | G3BP2 |
| SIRT1 | IL33 | AURKC | NQO1 | AHSG | PDLIM7 | FAM83D |
| PLK1 | TNFRSF10A | NGFR | HSPA8 | MAP1B | RPLP2 | HEMGN |
| PANX1 | TNFRSF10B | FMR1 | IKBKG | PPM1B | RPS16 | HSPBAP1 |
| DDX58 | FAF1 | GNLY | BDNF | PKM | RPLP1 | GOLGA3 |
| TARDBP | IFNA1 | HTRA2 | XBP1 | PABPN1 | TPM4 | HRNR |
| SLC39A7 | IFNA2 | HSPA5 | BECN1 | PKP2 | SRSF9 | GLTP |
| MAP3K7 | IFNA4 | PRKAA2 | SOX17 | ULK1 | SRSF1 | NUDT21 |
| SQSTM1 | IFNA5 | PRKAA1 | TAB1 | XRCC6 | CCNT1 | SERBP1 |
| OTULIN | IFNA6 | PITPNA | UBC | XRCC5 | DYNLL1 | SEC16A |
| FLT3 | IFNA7 | METTL3 | TAB3 | UBE2L3 | DNAJA1 | SRRM2 |
| TLR4 | IFNA8 | FNDC4 | AGFG1 | NDUFA4 | DDX17 | SRP14 |
| HSPA4 | IFNA10 | FNDC5 | EIF2A | NRIP1 | CCT3 | SRSF10 |
| BNIP3 | IFNA13 | TXN | TNIP2 | NSUN2 | BCLAF1 | ESYT2 |
| RNF31 | IFNA14 | RALBP1 | MIB2 | OPTN | AGO2 | ERH |
| BCL2 | IFNA16 | TP53I3 | H1-5 | RPS20 | RPL23A | MLF2 |
| CDC37 | IFNA17 | PRKN | DIRAS3 | STK38 | KRT86 | RBM14 |
| LEF1 | IFNA21 | GSDMD | C20orf204 | SFPQ | NME8 | PRPF40A |
| BCL2L11 | IFNB1 | NFKB1 | HMOX1 | TUBB4B | HNRNPH1 | POF1B |
| DIABLO | IFNG | RELA | MMP13 | LITAF | HNRNPL | VBP1 |
| CDKN2A | IFNAR1 | TRPC6 | NPM1 | RPL15 | HOOK1 | YTHDC1 |
| BRAF | IFNAR2 | SIRT5 | G6PD | KRT10 | HNRNPH3 | AKAP8L |
| AXL | IFNGR1 | MIR21 | GJA1 | LGALS1 | FIP1L1 | AKNA |
| MYCN | IFNGR2 | AURKA | TSC2 | KLF6 | PDIA6 | TRAFD1 |
| ALK | JAK1 | MYH9 | VIM | KRT16 | RPL6 | CALML5 |
| ATRX | JAK2 | CASP10 | CAD | KRT7 | RIOK1 | PNN |
| TERT | JAK3 | SOAT1 | DRD2 | MYO1C | RPL23 | ELP1 |
| BACH2 | TYK2 | HGF | ATP2A1 | EEF1A1 | RPL28 | KCTD5 |
| GATA3 | STAT1 | SRC | KRT18 | HNRNPU | RPL29 | RBM25 |
| CD40 | STAT2 | ANXA1 | RPS19 | FLII | SSBP1 | UBL4A |
| USP22 | STAT4 | FPR1 | TNNT2 | ACTC1 | SF1 | APOOL |
| TNFRSF1A | STAT5A | CASP2 | TPM1 | KHDRBS1 | TRA2B | CRTAM |
| TNFSF10 | STAT5B | MIF | EPAS1 | PNKD | TRA2A | CTAG2 |
| MYC | STAT6 | KL | APC | PSMA3 | SYNCRIP | LRRC59 |
| DNMT1 | IRF9 | BCL2L1 | AHR | RPL7A | SRSF7 | PPP1R12C |
| ID1 | EIF2AK2 | BMI1 | ACTB | RPS14 | CCT8 | PALMD |
| STUB1 | TICAM2 | BBC3 | LRP1 | RPS17 | ADAMTSL4 | TLE6 |
| SIRT3 | TICAM1 | AVEN | VCP | RPS24 | CCT6A | ZSCAN20 |
| KLF9 | USP21 | ZNF7 | YWHAE | RPS27 | DNAJA2 | ZKSCAN4 |
| HDAC9 | HSP90AB1 | PTEN | YWHAG | RPS3 | BAG2 | H1-2 |
| APP | TNFAIP3 | ESR2 | NOD2 | RPS6 | ATAD3A | RPL39 |
| TNFRSF21 | PARP1 | GSK3A | PIKFYVE | RPL13A | RCC2 | SP6 |
| STAT3 | BID | C5 | EEF2 | RPL13 | RFWD3 | CHTOP |
| CXCL8 | AIFM1 | SP1 | MAPKAPK2 | TANK | WDR77 | UBAP2 |
| HMGB1 | H2AX | EGR1 | PRDX1 | TCOF1 | LARP1 | ZAN |
| TSC1 | H2AC20 | C7 | KIF11 | TNFRSF8 | MYL12A | GSDME |
| TRIM11 | H2AC12 | C9 | SLC16A1 | TRIM28 | MYO1D | TMEM44 |
| IDH1 | H2AC1 | C6 | KRT8 | TUBA1C | MYO1B | CEP44 |
| IDH2 | H2AW | ATG5 | KRT5 | S100A6 | MYCBP | ATP5F1C |
| EGFR | H2AB3 | CD74 | KRT1 | TCP1 | HNRNPM | LZTS3 |
| HSP90AA1 | H2AC8 | NOX4 | KRT14 | SLC30A9 | MYL6B | RHOXF2 |
| NLRP3 | H2AC4 | TNFSF12 | HIF1A | CALM3 | GOSR1 | TMEM263 |
| TRADD | MACROH2A2 | MKRN1 | HSPD1 | ASIC1 | MRPS12 | ZNF391 |
| TRAF5 | MACROH2A1 | RCN1 | HSPA9 | CADM1 | RPS18 | H1-10 |
| XIAP | H2AC19 | UHRF1 | PINK1 | CALU | RPS8 | UTP11 |
| SHARPIN | H2AJ | AIFM2 | RPL5 | RBMX | RPS4X | H2BC12 |
| SPATA2L | H2AB1 | SGK1 | TPM3 | RPL22 | RPL34 | OBI1 |
| CYBB | H2AC17 | PAK1 | TRAF3 | RPL27 | SCYL2 | MAIP1 |
| CAMK2A | H2AC18 | CDC7 | SLC25A13 | PCM1 | THRAP3 | RBM14-RBM4 |
| CAMK2D | H2AC11 | CERK | BAP1 | PABPC1 | TRIP6 | MIR137 |
| CAMK2B | H2AC21 | CTSB | DDX3X | COPA | TMOD3 | MIR148A |
