# Supplementary material for: Immune infiltration and a necroptosis-related gene signature for predicting the prognosis of patients with cervical cancer
Source: Front Genet. 2023 Jan 6;13:1061107. doi: 10.3389/fgene.2022.1061107 (PMC9852722; doi:10.3389/fgene.2022.1061107)
Supplement: Supplementary file 2 [file DataSheet1.ZIP › Supplemental files/Table S2.docx]

| **180 DENRGs** | | | | | | |
| --- | --- | --- | --- | --- | --- | --- |
| SLC25A13 | TRADD | FASLG | TLR4 | TERT | H2AC6 | SVIL |
| SLC25A5 | PYCARD | RPS25 | MMP13 | CHMP4C | IDH2 | S100A10 |
| CAPN1 | TNFRSF10A | TNNT2 | KIF11 | HPRT1 | SLC25A10 | ATAD3A |
| BID | MYH14 | KLF9 | IDH1 | PLK1 | IRAK1 | H2BC12 |
| HGF | TYK2 | CPSF3 | GLTP | NOD2 | H2AC21 | TPM2 |
| VIM | CAV1 | CD274 | TPM1 | TUBA1C | H1-5 | RPL10A |
| NSUN2 | CASP2 | EGR1 | TPM3 | KRT1 | PKP3 | KRT6A |
| AP2S1 | EZH2 | TNFRSF10B | PARP1 | KCTD5 | PRKN | VDAC1 |
| LTBP1 | AKNA | TNFSF10 | IQSEC1 | RFWD3 | KRT5 | IRF9 |
| CAMK2B | GATA3 | RPL5 | AHSG | PLA2G4F | RPS23 | MYCBP |
| EIF4B | PPIF | ZBP1 | CDKN2A | JMJD7-PLA2G4B | KRT16 | TNFRSF25 |
| CASP8 | MAPK10 | POF1B | GSN | FASN | KRT14 | TNF |
| PKM | RPL34 | PPP1R12C | CCT5 | KRT8 | H1-2 | TNFSF12 |
| PYGM | RPS13 | TRAF2 | BCL2L11 | KRT86 | EIF4EBP1 | MIF |
| CAMK2A | KRT18 | TUBA4A | OTULIN | STAT2 | PLA2G4E | PLA2G4B |
| TCOF1 | IFNG | VIL1 | TRIM11 | CDC42BPG | TUBB4B | PGAM5 |
| TP63 | BACH2 | FLNC | EEF1A1 | KRT19 | H2AX | IKBKE |
| HSP90AA1 | BAG2 | MRPS12 | BUB1B | KRT9 | SP6 | TXNIP |
| NOX4 | C7 | MYO1B | IGF2BP1 | BCL2 | PPIA | H2AC12 |
| BAX | CLINT1 | DNMT1 | ACTC1 | CLEC7A | ATP2A1 | UHRF1 |
| AURKA | MACROH2A1 | MPRIP | PLA2G4D | RARG | C20orf204 | H2AC14 |
| SIRT1 | IL1A | HOOK1 | FNDC5 | STAT5B | MYO6 | H2AC16 |
| CDC7 | STAT1 | ANXA1 | ALOX15 | RPL15 | H2AC13 | H2AC8 |
| LGALS1 | RPL22 | KRT7 | AIM2 | BDNF | H2AC11 | H2AC17 |
| RPL3 | CAPZA1 | IGF2BP3 | RPL9 | CALML5 | H2AC7 |  |
| FAM83D | IVNS1ABP | TXN | H2AZ1 | RCC2 | PDLIM7 |  |
