# Supplementary figures and images for: Immune infiltration and a necroptosis-related gene signature for predicting the prognosis of patients with cervical cancer
Source: Front Genet. 2023 Jan 6;13:1061107. doi: 10.3389/fgene.2022.1061107 (PMC9852722; doi:10.3389/fgene.2022.1061107)

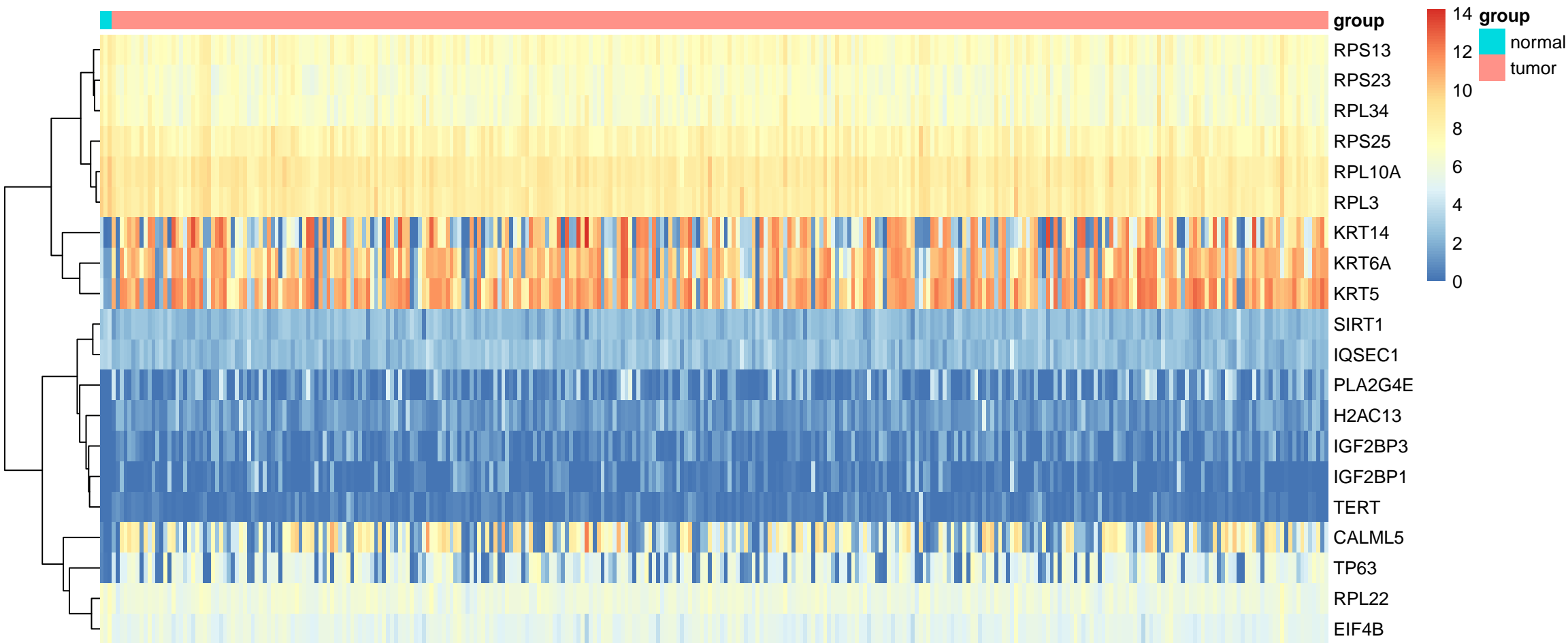

Supplement: Supplementary file 2 [file DataSheet1.ZIP › Supplemental files/Figure S1.pdf]

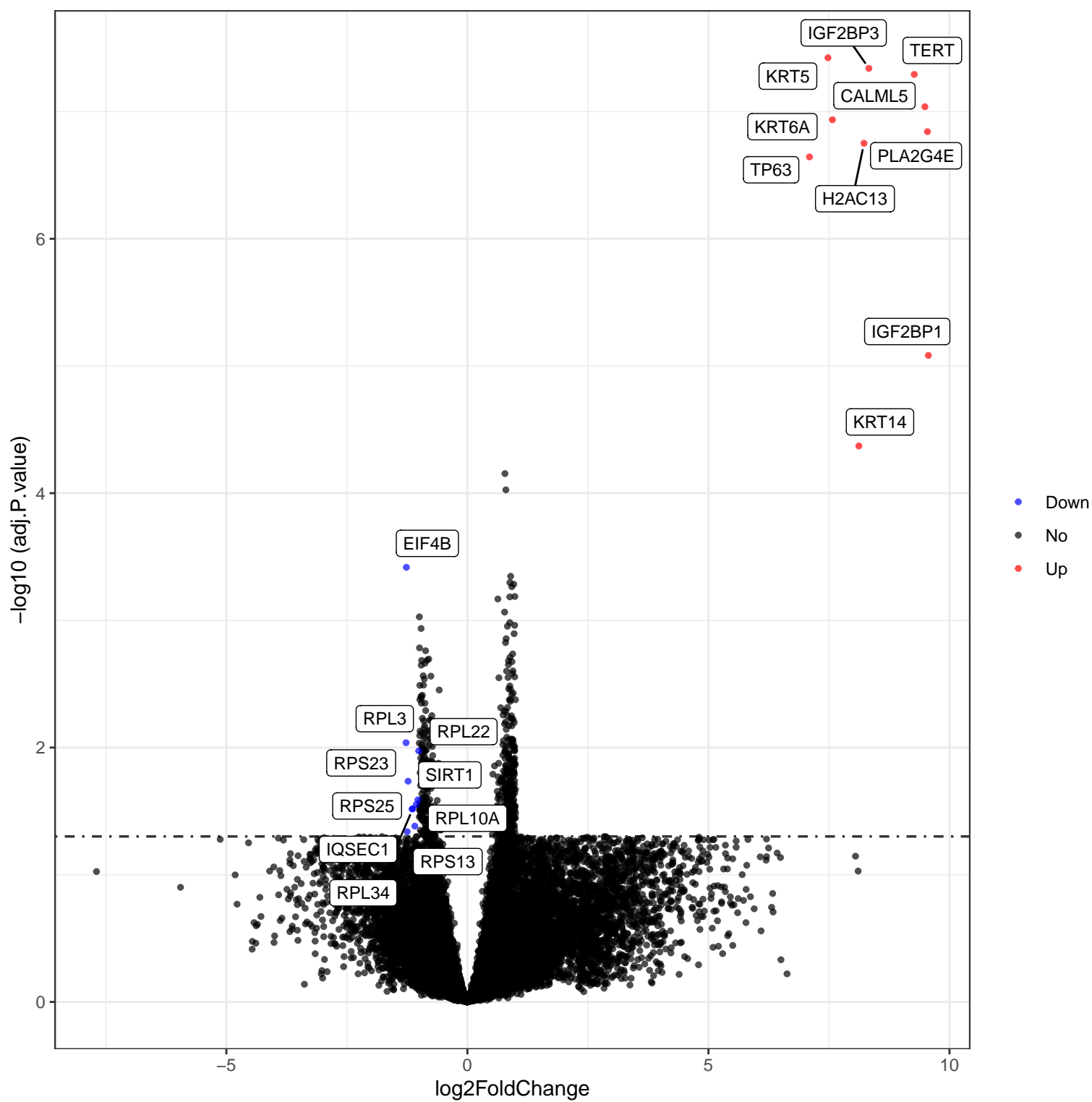

Supplement: Supplementary file 2 [file DataSheet1.ZIP › Supplemental files/Figure S2.pdf]

Variable

M1vsM0

N1vsN0

T4vsT1

T3vsT1

T2vsT1

Age

RiskScore

0

5

10

15

Harzard Ratio

p.val

0.6

0.4

0.2

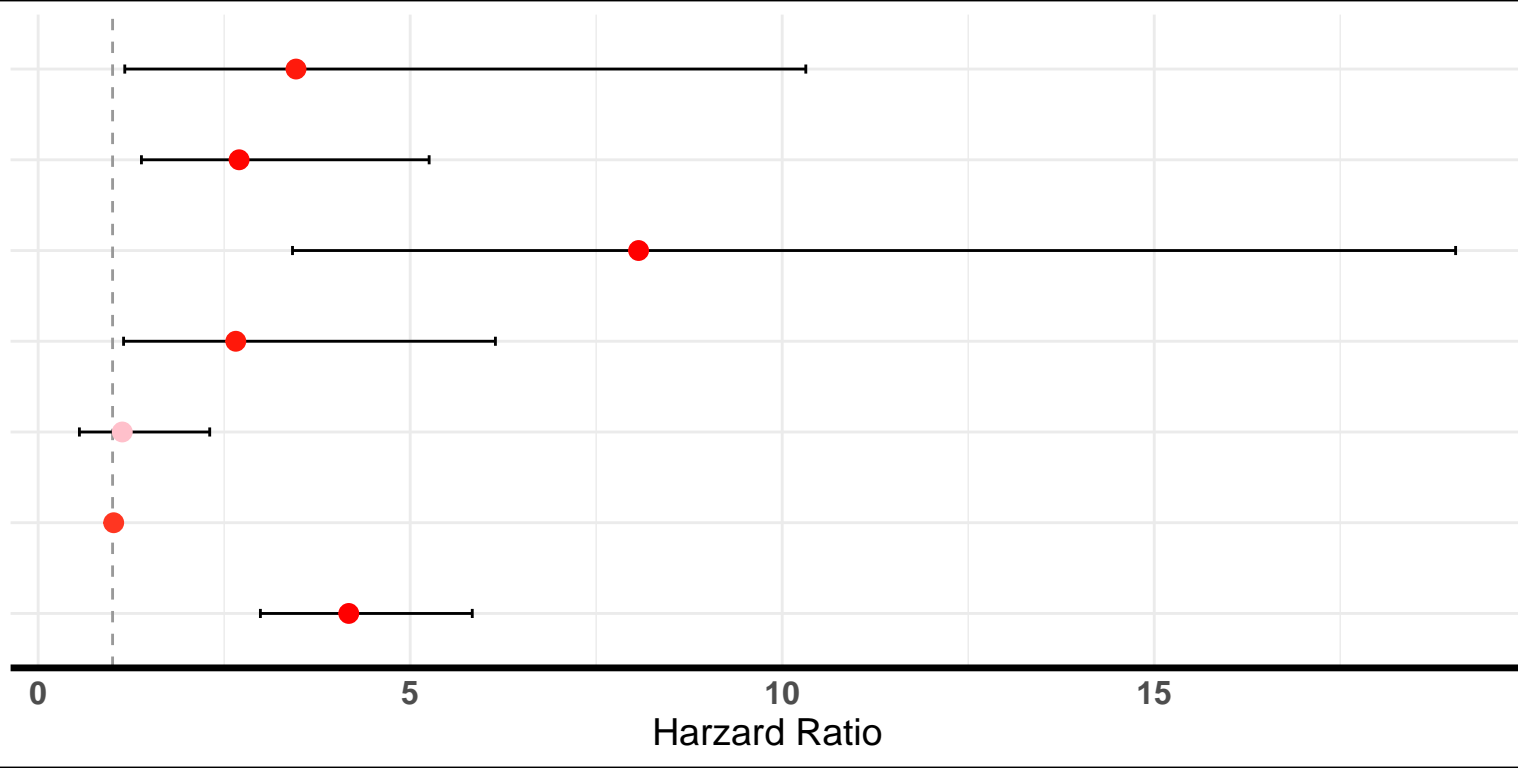

Supplement: Supplementary file 2 [file DataSheet1.ZIP › Supplemental files/Figure S3.pdf]
