# Supplementary material for: Immune infiltration and a necroptosis-related gene signature for predicting the prognosis of patients with cervical cancer
Source: Front Genet. 2023 Jan 6;13:1061107. doi: 10.3389/fgene.2022.1061107 (PMC9852722; doi:10.3389/fgene.2022.1061107)
Supplement: Supplementary file 6 [file DataSheet2.DOCX]

The datasets supporting the conclusions of this article are available in The Cancer Genome Atlas (TCGA) , GSEA-msigDB databases and additional files.

| **software** | **version** |
| --- | --- |
|  |  |
| GSVA | 1.1.0 |
| limma | 3.46.0 |
| clusterProfiler | 3.18.1 |
| pathview | 1.30.1 |
| enrichplot | 1.10.2 |
| VennDiagram | 1.6.20 |
| ggplot2 | 3.3.5 |
| ggpubr | 0.4.13 |
| beeswarm | 0.4.0 |
| Corrplot | 0.90 |
